# Cytokinin Translocation to, and Biosynthesis and Metabolism within, Cereal and Legume Seeds: Looking Back to Inform the Future

**DOI:** 10.3390/metabo13101076

**Published:** 2023-10-13

**Authors:** Paula E. Jameson

**Affiliations:** School of Biological Sciences, University of Canterbury, Private Bag 4800, Christchurch 8140, New Zealand; paula.jameson@canterbury.ac.nz

**Keywords:** cytokinin, seed, legume, cereal, zeatin, IPT, CPN1, CYP735A

## Abstract

Early in the history of cytokinins, it was clear that *Zea mays* seeds contained not just *trans*-zeatin, but its nucleosides and nucleotides. Subsequently, both pods and seeds of legumes and cereal grains have been shown to contain a complex of cytokinin forms. Relative to the very high quantities of cytokinin detected in developing seeds, only a limited amount appears to have been translocated from the parent plant. Translocation experiments, and the detection of high levels of endogenous cytokinin in the maternal seed coat tissues of legumes, indicates that cytokinin does not readily cross the maternal/filial boundary, indicating that the filial tissues are autonomous for cytokinin biosynthesis. Within the seed, *trans*-zeatin plays a key role in sink establishment and it may also contribute to sink strength. The roles, if any, of the other biologically active forms of cytokinin (*cis*-zeatin, dihydrozeatin and isopentenyladenine) remain to be elucidated. The recent identification of genes coding for the enzyme that leads to the biosynthesis of *trans*-zeatin in rice (*OsCYP735A3* and *4*), and the identification of a gene coding for an enzyme (CPN1) that converts *trans*-zeatin riboside to *trans*-zeatin in the apoplast, further cements the key role played by *trans*-zeatin in plants.

## 1. Introduction

The cytokinins are considered a key driver of seed yield, being implicated in both seed number and seed size, which are core agronomic traits in crop plants that affect crop yield, e.g., [1,2,3,4,5]. The history of research into the cytokinins in seeds is inextricably linked with the identification of zeatin and its conjugates. Zeatin was identified from *Zea mays* seeds by Letham and colleagues in 1964 [6]. The review ‘Zeatin: the 60th anniversary of its identification’ [7] did not include a detailed exposition of early research into the translocation of cytokinins to and within pods and seeds, or the metabolism of cytokinins during seed development. This review considers these aspects and introduces more recent work as it validates, or not, the conclusions from what was the era of biochemical analyses of legumes and cereals.

This discussion is restricted to cereals, which are endospermic seeds, destined to store metabolites within the endosperm, and legumes, which are non-endospermic seeds, wherein the cotyledon performs that role [8]. Following fertilisation, all seeds undergo a short syncytial phase of free nuclear divisions [9]. However, development diverges with the development of cereal endosperms progressing rapidly through the coenocytic nuclear divisions, mitotic divisions and cellularisation. During cellularisation, endosperm transfer cells, the aleurone and starchy endosperm differentiate [10] and storage product accumulation subsequently occurs. The metabolites and storage products accumulated in the cereal endosperm support the embryo during both embryogenesis and seed germination [9].

In legumes it appears that the endosperm remains rather fluid [11,12,13], supporting the morphogenesis phase that involves rapid cell division followed by a period of cell expansion. The seed (seed coat, endosperm plus incipient embryo) reaches full size before major development of the embryo. In eudicots, the embryo is normally the major part of the mature seed, and the bulk of the endosperm tissue is often ephemeral, degrading with the component nutrients transported and utilised by the embryo [8,14,15]. The cotyledons become the site of storage product accumulation and they support germination. 

During the 1950s and early 1960s, multiple laboratories were actively working to identify naturally occurring cell-division-inducing factors [7]. Early research by Letham drew positive correlations between cell-division-inducing factors in bioassays and cell division in apple [16] and plum fruitlets [17]; both cell-division-inducing factors were likely to be *trans*-zeatin (*t*Z). However, Miller [18] was the first to monitor cell-division-inducing activity across development in seeds, finding little cell-division-inducing activity in maize ovules extracted on the day of pollination or even three days later. Most activity was detected at 6 DAP (likely *t*Z) and 11 DAP (likely *t*Z and nucleotide(s)) but this had reduced by 21 DAP, with no activity detected in mature grains [18]. Not surprisingly, much of the early research with the cytokinins was focused on their cell-division-inducing activity. However, Mothes and Engelbrecht [19] also showed the marked ability of cytokinins (kinetin in this case) to attract metabolites to their site of application, raising the possibility of cytokinins being key factors in hormone-directed transport, independent of their cell-division-inducing activities [20]. Both of these activities are currently the subject of some debate. This review considers the complex nature and biological activity of the cytokinins identified in seeds, whether seeds are autonomous for cytokinin production and whether this biosynthesis occurs in maternal and/or filial seed tissues, and the role of cytokinins in sink establishment and sink strength during seed development. 

## 2. The Complex of Cytokinins within Cereal Kernels, and within Pods and Seeds of Legumes

### 2.1. Cereals

Zeatin (*t*Z: the original name refers to the *trans* isomer) was the first naturally occurring cytokinin to be identified from any plant tissue [6]. Additional biologically active components were also present in *Z. mays* kernels. As zeatin is an amino purine, both riboside and nucleotide forms were predicted to exist [21,22]. Both the riboside (ZR) and nucleotide (ZRMP) of *t*Z were unequivocally identified in *Z. mays* kernels [23,24,25]. Subsequently, two additional free bases, dihydro-Z (DZ), isopentenyl adenine (iP), their ribosides (DZR, iPR), and nucleotides (iPRMP, DZRMP), the *O*-glucosides of tZ, DZ, ZR and DZR, and the 9-glucosides of *t*Z (Z9G), DZ, *c*Z and iP have been identified [26,27,28,29,30,31]. Suggestions of the presence of *cis* derivatives in kernels [22,25] were confirmed with the identification of *c*Z, *c*ZR, *c*ZRMP, *c*ZOG and *c*ZROG [32]. In total, 22 endogenous cytokinins have been identified in *Z. mays* kernels. 

With the exception of the 7-glucosides, all the known isoprenoid cytokinins in both *trans* and *cis* configurations have been identified in *Z. mays* kernels. The biosynthesis of cytokinins occurs via two pathways: the adenosine phosphate–IPT pathway leading to the *trans*-zeatin isomers, and the tRNA–IPT pathway, providing *cis*-isomers through tRNA degradation [33]. The nucleotides (NTs) formed via the adenosine phosphate–IPT pathway may be 5′-mono-, di- or tri-phosphates [34], which can be hydroxylated by specific cytochrome P450 (CYP) monooxygenases to form *t*ZNTs [35,36]. Cytokinin 5’-monophosphates are converted directly via LOG to the free base forms [37]. Zeatin reductase potentially reduces the *t*Z-types to the dihydro derivatives, although a gene coding for the reductase has not yet been identified [31]. The free bases *t*Z, *c*Z, DZ and iP are considered the biologically active forms on the basis of binding to receptors [38,39], with *t*Z, *c*Z and iP and their ribosides susceptible to degradation by cytokinin dehydrogenases (CKX; [40]). The *O*-glucosides are inactive per se and resistant to CKX but are able to be reactivated via β-glucosidases. The 7- and 9-glucosides of Z are generally considered deactivated forms but may contribute to the active cytokinin pool, whereas the 7- and 9-glucosides of iP are considered terminal metabolites (see reviews by [7,41]). 

Cytokinin ribosides have been identified as a key transport form in the xylem and an enzyme capable of converting riboside cytokinins to free base cytokinins was very recently described by Kojima et al. [42]. The cytokinin/purine riboside nucleosidase 1 (CPN1) enzyme was located at the cell wall and shown to deribosylate cytokinin ribosides, including *t*ZR, *c*ZR, *t*ZROG and *c*ZROG. Purine nucleosides, such as adenosine, inosine and guanosine were also deribosylated [42]. Consequently, *t*ZR and *c*ZR arriving in the apoplast via the xylem can be converted to their free base active form, and either detected by receptors on the plasma membrane or translocated into the cytosol [42] (see their Figure S11 for a schematic model of the involvement of OsCPN1 in apoplastic cytokinin metabolism). 

Complexes of cytokinins similar to those identified in maize are found in other cereals. In wheat (*Triticum aestivum*), early bioassay work suggested a complex of bioactive cytokinins and glucosides with zeatin predominant, e.g., [43,44,45]. Recent comprehensive LC-MS/MS analyses revealed the presence of 20 isoprenoid cytokinins, with a predominance of *t*Z- and *c*Z-type cytokinins and low levels of iP types and DZ types [46,47]. There is only one report of a 7-glucoside (*t*Z7G) in wheat [48]. The complex glucosides mentioned in [45] may have included the *t*Z9GOG and *t*Z-9-glucoside riboside reported by [48]. Z9GOG was also identified in young wheat spikes [49]. Additionally, complex *O*-glucosylated nucleotides have been identified as metabolites in rice [42,50], and a ring glucosyl form in barley [51], but have not been pursued in wheat. Low levels of four 2-methylthiol cytokinins (2MeSCK), derived from tRNA turnover [52], were identified alongside 21 conventional cytokinins [46]. 

In barley (*Hordeum vulgare*), some 20 isoprenoid cytokinins have been identified in grains, again with the *t*Z- and *c*Z-types predominating [47,53,54].

Cytokinins identified in rice (*Oryza sativa*) grains include the free bases *t*Z, *c*Z and iP; their ribosides and nucleotides; the *O*-glucosides of *t*Z and *c*Z; and Z9G. The *cis* isomers of ZRMP, ZROG and ZOG were also identified, which was likely their first identification in plant tissues [55,56]. Dihydro derivatives were not mentioned. 

### 2.2. Legumes

Many of the early endogenous cytokinin identifications were carried out on legumes. Dihydrozeatin from immature seeds of yellow lupin (*Lupinus luteus*) is likely the first unequivocally identified cytokinin in a legume [57]. High levels of multiple cytokinin types were subsequently detected by bioassay in white lupin (*Lupinus albus*) [12,58,59]. Concurrently, Z, DZ, ZR DZR and their *O*-glucosides were identified in pod walls and seeds of yellow lupin [60,61]. 

The GC-MS profiling of cytokinins in chickpea (*Cicer arietinum*) highlighted the presence of the *cis*-isomers of ZRMP, ZR and Z, which were present in greater concentrations than those of the corresponding *trans*-isomers or DZ. Levels of DZ-, DZMP- and iP-types were either low or undetectable [62]. 

Skoog’s lab was unsuccessful in identifying cytokinins from pea (*Pisum sativum*) (refer [7]). Subsequently, 10 cytokinins were initially identified [63,64], and some 21 cytokinins have now been identified by LC-MS/MS in pea pods and seeds, including the nucleotides, free bases and ribosides of both Z-type and iP-type cytokinins. Both *cis*- and *trans*-zeatin derivatives were identified along with low levels of dihydro derivatives of zeatin. *O*-glucoside forms and the 7-glucoside of zeatin were identified, but not the 7- or 9-glucosides of iP [65]. 

The profile of cytokinins in soybean (*Glycine max*) pods and seeds is equally as complex, with 22 forms (including three methylthio derivatives) identified across a range of cultivars [66,67,68]. The 7- and 9-glucosides of iP were not mentioned. 

### 2.3. cis Derivatives

The origin and biological relevance of the *cis* derivatives are of considerable interest. Initially thought to be of no consequence due to low activity in callus bioassays [69,70,71], and considered an artifact of extraction procedures [72], it came as a surprise when several publications indicated that the levels of *c*Z-isomers exceeded those of *t*Z-isomers at certain stages of development, including in both cereals and some legumes. Indeed, it has been suggested that the high levels of *cis*-isomers in many crop plants may be a result of the plant breeding process itself [73]. 

Contrary to the statement in [74], there is little evidence supporting the existence of the *cis-trans* isomerase reported by Bassil et al. [75] (e.g., [50,62,76,77]) and recent work suggests that the CYP735A pathway is likely the predominant pathway for *t*Z-type cytokinin biosynthesis in both monocots and dicots [36]. Lacking the identification of any other route, the origin of the *cis*-isomers appears to be tRNA degradation. 

While arabidopsis (*Arabidopsis thaliana*) receptors are essentially insensitive to *c*Z [38], it is clear that cytokinin-responsive histidine kinases (HK) in maize are responsive to *cis*-isomers [78]. Indeed, the sensitivity of ZmHK1 to *c*Z was comparable to that to *t*Z; in like manner, rice receptors OsHK3 and OsHK4 show a similar affinity to *c*Z and *t*Z [79]. However, the receptors in potato, which has an excess of *cis*-isomers relative to *trans*-isomers [80,81], are relatively insensitive to *c*Z [82], raising the question of the sensitivity of other eudicot receptors to *c*Z. Notably, while cytokinin receptors have been identified in several legumes (cited in [82,83]), none appears to have been assessed for their relative sensitivity towards *t*Z, *c*Z, DZ or iP. Such an investigation of legume receptors is warranted to determine whether it is only cereal receptors that are strongly activated by *c*Z.

Tracer experiments in rice showed that *c*ZR was metabolised predominantly to *c*ZOG, whereas *t*Z was metabolised predominantly to *t*Z-nucleotides, highlighting that *O*-glucosylation of *cis* derivatives to inactivated forms was marked [50]. However, the study concluded that *c*Z plays a role as an active cytokinin in several aspects of rice growth and development [50]. In contrast to this, Kiba et al. [36] recently suggested, in the absence of *t*Z-type cytokinins, *cis*-isomers play little role. Using double mutants of *Oscyp735a3* and *cyp735a4*, which prevent the hydroxylation of iP nucleotides to *t*Z nucleotides, they showed that levels of *cis* derivatives were unaffected, drawing the conclusion that the *cis* derivatives played little if any role in the growth-retarded phenotype of the double *cyp* mutants, where the levels of *t*Z-type cytokinins were much reduced [36]. 

The *cis*-isomers in cereals potentially accumulate because they can be actively *O*-glucosylated by *cis*-*O*-glucosyl transferases (OGTs), thereby conferring resistance to degradation by CKX [31]. *cis*-OGTs have been detected in maize [32,84], rice [50] and wheat [46,85]. Interestingly, the *O*-glucosyl transferase isolated from bean (*Phaseolus lunatus*) was specific to *t*Z [86]. Hluska et al. [31] make a pertinent observation, commenting that even though equal amounts of *c*Z originate continuously in all species by the same house-keeping process of tRNA isopentenylation and subsequent RNA decay, *c*Z can preferentially accumulate in species like maize due to the action of *c*Z-specific OGTs, as *c*ZROG is protected against degradation by CKX enzymes, whereas *t*Z9Gs are not so protected [31,87]. Moreover, side-chain cleavage, rather than side-chain modification, appears to be the dominant form of *t*Z metabolism with minimal *O*-glucosylation, at least in mature maize kernels [27]. 

### 2.4. Complexity Issues

In summary, legume pods and seeds and cereal grains contain complexes of cytokinins, as both *trans* and *cis* isomers. The 7-glucoside of zeatin, first identified as an endogenous cytokinin in radish seed [88] and a prominent cytokinin in arabidopsis and tobacco (see discussion in [89]) has not been routinely identified in either cereals or legumes, nor have the 7- or 9-glucosides of iP. However, clearly there are multiple different cytokinin forms in cereals and legumes, providing evidence of active cytokinin metabolism. Such complexity does not lead to simple analyses. Immunoassays have been used to successfully analyse cytokinin complexes, but samples require comprehensive purification and separation of the various cytokinin forms [29,30]. Simple reliance, as in recently published papers, on purchased kits can lead to misleading results (e.g., [90]). 

Another issue that has led to questions about the role of the cytokinins in developing seeds is the correlation of cytokinin type and/or amount with stages of development, and the analyses of mixed or separated tissue types. Consequently, in the analyses discussed below, developmental information is provided where known.

## 3. Sites of Cytokinin Biosynthesis during Pod and Seed Development and Maturation

Clearly cereal grains and pods and seeds of legumes contain a wide range of cytokinins, but the source and the role of the cytokinin is the subject of debate. Early bioassay-based experiments led to different conclusions. For example, based on cytokinin flux in bleeding sap, roots were considered as the potential supplier of cytokinin to *Perilla frutescens* fruits [91], whereas the seed was considered the potential source of cytokinin for apple fruit [92]. However, in a critical analysis of multiple experiments, Letham [93] concluded in 1994 that root tips were a site of cytokinin biosynthesis, whereas shoots, with the exception of seeds, were largely dependent on root-supplied cytokinin. As shown in Sakakibara et al. [3], recent models depict *t*Z biosynthesis to be mainly localized in the root tissues, while iP-type cytokinins are produced both in roots and in aerial plant parts. This notion has been based on the expression of arabidopsis *CYP735As*, which is predominantly in the roots [35]. However, Kiba et al. [36] show rice *CYP735A4* is also located in shoot tissues, including low levels in panicles, providing shoot-based sites of *t*Z synthesis in rice.

An early experiment purported to show cytokinin biosynthesis in seeds [94]. Intact pea pods isolated 10 days after anthesis were grown in vitro and showed not only that the peas grew to maturity, but also that they were able to germinate. The cytokinin content, measured by bioassay, increased up to 12–18 days in culture, independent of the presence of roots [94]. In contrast, two other groups could not validate this claim as neither could detect biosynthesis of cytokinin in pea fruits cultured in vitro [95,96]. 

Following the identification of a very high level of DZROG in the pod walls of yellow lupin, the comment was made that the reported increase in cytokinin content of the pea seeds [94] may only reflect import of cytokinins from pod walls due to the release of active cytokinins from DZROG [60]. Consequently, the evidence that pea seeds synthesize cytokinins required re-evaluation [60]. In investigating this further, seeds of yellow lupin were removed from pods about five days after anthesis (DAA) and incubated with ^3^H-adenosine [61]. Radioactively labelled ZOG and ZROG were subsequently detected, confirming that the isolated yellow lupin seed was capable of cytokinin biosynthesis [61], at least in vitro.

While expression of adenylate-IPT, the committed step in cytokinin biosynthesis, has been shown in precise regions throughout the plant and in developing seeds of arabidopsis [97], as well as in seeds of a number of other species (refer [4]), it is also without question that cytokinin movement occurs in the plant in both xylem and phloem (e.g., [3,12,42,93]), and could be a source of cytokinin for developing legume fruits and cereal grains. 

### 3.1. Legumes

#### 3.1.1. Source of Cytokinin in Legumes

While a range of cytokinins has been identified from both the phloem and xylem sap of legume species, work with labelled cytokinins in a variety of tissues and species indicates that *t*ZR is a key transport form in the xylem (see discussion in [7,34]).

Much of the early work from Letham and co-workers involved the translocation and metabolism of radioactively labelled cytokinins in legumes [7]. Work with soybean and various lupin species utilised de-rooted plants, explants, and intact plants where the cytokinin was supplied via a wick to the xylem. In terms of pod and seed development, a critical finding showed that while supplied adenosine readily reached the soybean embryo, similarly supplied cytokinin did not [98]. Only limited quantities of cytokinin reached the pod wall and seed coat of soybeans and blue lupins (*L. angustifolius*) [98,99,100,101], with the conclusion being that the seeds did not compete with leaves for xylem supplied cytokinin and that cytokinin did not readily cross the apoplastic space between the maternal seed coat and the filial embryo, implicating synthesis in situ. 

The recent suggestion by Radchuk et al. (2023) [102], that certain SWEETs have the ability to move both sugar and cytokinin across the maternal/filial boundary during seed fill in barley, does not seem to be apply to the movement of cytokinins in legumes under the conditions of the above experiments. 

To determine contributions from phloem sap, white lupins have been used as they readily yield phloem exudate (e.g., [103]). When ^3^H-ZR was supplied to roots of white lupin and the phloem sap collected from the top of the pods, the principal metabolite was DZ [61], leading to the conclusion that the cytokinin moved through the phloem of the pod wall [61]. Analysis of phloem sap exuded from the base of the inflorescence and base and tips of white lupin pods showed (by bioassay) that sap cytokinin levels increased as fruit developed, with ZR and DZ identified by GC-MS as major constituents of a combined phloem and xylem sample [104]. 

However, following a comprehensive GC-MS analysis of cytokinins in phloem, xylem and developing white lupin fruits, Emery et al. [13] concluded that, while translocated cytokinins from phloem and xylem could account for the cytokinin requirements during early pod set, the increases in cytokinin in the seed coat and endosperm indicated synthesis in situ. Similarly, Zhang and Letham [101] concluded that xylem-derived cytokinins may exert little if any control over embryo development in legume seeds, which may be completely autonomous in terms of cytokinin. 

#### 3.1.2. Sites of Cytokinin Accumulation within Legume Pods and Seeds

In lupins, the pod walls reach maximum length and width before any significant development of the embryo within the pod. Seed enlargement begins after fertilisation with a rapid increase in size as cells divide and expand, but with little increase in seed weight for about 5.5 to 6 weeks after pollination. During the pre-storage phase, endosperm development, cell division and embryo differentiation occur. The seed coat and endosperm develop first, followed by the development of embryo [105]. As seed fill commences, cell division has stopped, cotyledon cells continue to expand as they take in water and nutrients and seed dry weight increases with the rapid accumulation of storage reserves. Seed fill is completed by about 12 weeks (from Lupin Growth and Development: www.industry.nsw.gov.au). Other legumes develop faster (peas) or slower (chickpeas) than the description for lupins above [106]. In legumes, generally, the size of the seed is considered to be primarily associated with the initial growth of the endosperm and not with the later growth of the embryo [107].

##### Chickpea

A comprehensive GC-MS identification and quantitation of cytokinins during chickpea seed development was carried out, with pods collected at four stages of development: at 1, 14, 30 and 40 days after pod set (DAPS), with pods 3 mm long regarded as showing pod set [62]. Cytokinins were extracted from whole pods (fertilized ovaries, 1 DAPS, start of cell division); from embryo, endospermic fluid and seed coat at 14 DAPS (end of cell division and commencement of seed filling); at the maximum rate of seed filling (30 DAPS); and at the end of seed filling (40 DAPS). Pods harvested at 14 DAPS were partitioned into pod wall, seed coat, embryo and endospermic fluid. Pods harvested at 30, 40 and 50 DAPS were partitioned into pod wall, seed coat, cotyledons and embryonic axis [62].

Cytokinin profiles were relatively consistent across seed tissues and stages of development. With the exception of iPRMP, which was never detected, the cytokinin nucleotides were present in the greatest concentrations, followed by the ribosides and free bases, respectively. iP and iPR were present in low but detectable quantities only in older cotyledon extracts from seeds in which cell division had ceased. Generally, levels of *c*ZRMP, *c*ZR and *c*Z predominated over their corresponding *trans* isomers, except for the embryo at 14 DAPS, where *t*Z predominated and no *c*Z was detected. The concentration of total cytokinin was highest in the endospermic fluid as cell division ended at 14 DAPS, noting that no analyses were carried out between 1 DAPS and 14 DAPS to determine the timing of ‘peak’ cytokinin. During rapid seed filling at 30 DAPS, the concentration of total cytokinin had decreased considerably and continued to decline to a low by the end of seed filling at 40 DAPS. While concluding that their data demonstrate that the cytokinin levels in chickpea are greatest over the phases of rapid cell division [62], their analyses did not include the phase of free nuclear divisions, occurring immediately post fertilisation.

##### Lupins

Analyses of dissected pods, seed coats, endosperm and cotyledons of lupins reveal a complex pattern of endogenous cytokinins. Careful bioassay work indicated the presence of free bases, glucosides and a nucleotide [12,58,59], with total cytokinin activity elevated in both pods and seeds of white lupin [58]. Closer analysis of dissected seeds showed, at the peak of activity six weeks after anthesis when the endosperm filled most of the seed cavity, the highest levels of bioassay-active cytokinins were in the endosperm relative to the seed coat and embryo. By eight weeks after anthesis the cotyledons filled the seed cavity, by which time the level of cytokinin in the seed coat exceeded both that in the remaining endosperm and cotyledon [12].

Seeds and pod walls of yellow lupin were extracted 14 days after petal fall [60]. ZOG, DZOG, ZROG, DZROG, ZR and DZR were identified and quantified in both seeds and pod walls (except ZR in pod walls). DZROG was at noticeably greater levels in the pod walls than other cytokinins, while DZROG, ZR and DZR were at similar levels in the seeds. The levels of cytokinins were considerably greater in developing seed than in seed approaching maturity. ZR, DZR and DZROG were the dominant cytokinins in the developing seed, while DZR and DZROG were the dominant cytokinins in seed approaching maturity (24 days from petal fall) [61].

Blue lupin seeds were dissected into seed coat and embryo 30 days after flowering (DAF), by which time the liquid endosperm had disappeared and the seed coat to embryo FW ratio was 2.56 [101]. Some 83% of the seed-contained cytokinins were reported to be in the seed coat (1041.8 versus 219.3 pmol/g FW for embryo quantified by RIA). The authors commented that it was “unexpected” to find the high level in the seed coats relative to embryos and suggested that “seed coats of legume seeds appear to shield the embryos from the low proportion of xylem cytokinins that may reach the seed” [101]. The majority of cytokinins were of the DZ type, particularly DZR, DZNT and DZ in the seed coats, with lesser levels of these forms in the embryo. Free bases Z, DZ, iP and iPNT were all detected, as were the *O*-glucosides. Each of the cytokinin forms was at a greater level in the seed coats [101]. *cis*-Isomers were not mentioned. 

Comprehensive GC-MS analyses of white lupin were initiated at anthesis and included pod set, cell division, morphogenesis and seed filling up to physiological maturation 77 DAA [13]. In the first 10 DAA, the fertilized ovaries destined to set pods (position 1) accumulated cytokinin, with the proportion of *cis*- to *trans*-isomers initially at 10:1 but declining to less than 1:1 by 10 DAA in those destined to set pods, whereas in ovaries destined to abort (position 3) the ratio of *cis*- to *trans*-isomers remained high [13]. However, a closer look showed that cytokinins considered today to be biologically active forms, *t*Z or *c*Z, were not reliably detected whether pods were destined to set or not. The developing ovaries at all positions accumulated *c*ZRMP, the major cytokinin identified in the phloem and xylem exudate, but also accumulated *c*ZR at a much higher relative ratio than that supplied from the phloem and xylem. Notably, while DZRMP was detected in all ovary tissues, *t*ZRMP, the immediate precursor of *t*Z, was detected at anthesis only in those pods destined to set (position 1). 

As the white lupin seed continued to develop, pod wall cytokinins remained low relative to those accumulating in the seed coats and endospermic fluid [13]. During embryogenesis and early seed filling, the quantity of *t*ZR in the endospermic fluid increased 10-fold from between 40 and 46 DAA to a massive 239,414 pmol/g FW, predominantly as *t*Z- or dihydro-derivatives. Multiple forms were detected at elevated levels: *t*ZR > *t*ZROG >DZR >> iPR~*t*Z~*t*ZOG~*c*ZR > DZ~*c*ZROG~*t*ZRMP > DZRMP > *c*ZRMP (totalling some 620,994 pmol/g FW). Peak cytokinin levels in seed coat (13,748 pmol/g FW) and cotyledon (17,770 pmol/g FW) also occurred at 46 DAA and, while considerably lower, generally mirrored those in the endosperm in terms of type, but also included DZROG in the seed coat. Once the endosperm had been subsumed, the high cytokinin concentrations in the seeds declined rapidly in the cotyledon, but somewhat less rapidly in the seed coats, where elevated levels of DZR > DZRMP > DZ were still detectable at physiological maturity [13]. [The high cytokinin levels are reported in the text as >0.6 mmol/g FW, potentially 1000-fold unit error; author calculates this to be >0.6 µmol/g FW].

The GC-MS data on white lupin [13] confirms the extraordinarily high cytokinin levels in the endosperm of white lupin detected by bioassay [12], which were challenged [101]. 

##### Soybean

Based on cytological studies of soybean embryogenesis, cell division in the seed is completed at an early stage of development (R4) while the embryo is still quite small. The major increase in seed size occurs from the beginning of R5 to the end of R6 and through enlargement of pre-existing cells (reviewed in [108]). The total concentration of cytokinins quantified by RIA, following exhaustive separation procedures, at mid to late podfill in soybean seed coats was considerably greater than that in the cotyledons [100]. The dominant cytokinins in the seed coats were DZ, its nucleotide (DZRMP) and iPRMP, while in the cotyledon *t*Z predominated. The *O-*glucoside, DZROG, was prominent in seed coats but was not detected in cotyledons [100]). 

Fourteen cytokinins were identified using LC-MS/MS in soybean tissues [66]. Full pods (R4 stage), ranging from 10–20 mm in length, were collected. No *cis* derivatives were detected in the pods, while *t*Z, DZ, iP, their ribosides and nucleotides were identified. The hydroxylated cytokinins (*t*Z and DZ types) greatly exceeded those of the iP types, with *t*ZR and DZR contributing the most in the pods. *GmtRNA-IPT2* was highly and ubiquitously expressed across a range of tissues, yet few *cis* derivatives were detected in any tissue [66]. *GmIPT1* and *tRNA-IPT2* expressed in the greatest abundance in full pods, while *GmIPT1*, *11* and *tRNA-IPT2* were the most abundant transcripts in R5 seeds, particularly *IPT11*. The variation in the *GmIPT* transcript levels in flowers, pods and R5 seeds indicated that each of these organs required different *GmIPT* genes for cytokinin biosynthesis [66]. However, in this analysis, intact seeds were used, which included the seed coat, preluding determination of the site(s) of cytokinin synthesis within the seed.

An extensive LC-MS analysis of cytokinin levels in 27 field-grown soybean cultivars was conducted [67]. Pods at two stages of development were analysed: [R4 (full pod) at 20–30 DAF, and R5 (beginning seed; ⅛ inch long) at 30–45 DAF]; and seeds at R6 [full seed; green seed fills pod cavity] at 45–65 DAF [67]. In whole pods at R4, DZR levels > *c*ZR >> *t*ZR~iPR and *t*Z~*c*Z > DZ levels, while iP levels were always lower. In terms of nucleotides, *c*ZNT > iPNT > DZNT~*t*ZNT, with a similar pattern in R5 pods. Correlations between the 14 cytokinins detected and various yield parameters were investigated. Considerable variability can be seen among the cultivars and significant positive correlations between yield components and *t*Z were detected for both R4 and R5 pods. Interestingly, a similar trend was reported for *c*Z [67]. Seeds were separately analysed at one growth stage. The authors suggest a switch in hormone profiles had occurred as Z type cytokinins were not detected, but there were increased levels of iP types in high-yielding cultivars. The authors suggested that the presence of iP derivatives allowed developing seeds to maintain their active role in sink organs and to attract assimilates during the seed filling phases. The methylthiols, especially MeSZ, were also positively correlated with yield across all three reproductive growth stages [67].

More recent research from the same lab [68] used four greenhouse-grown cultivars but only one of the same cultivars from the trial above [67]. Comprehensive LC-MS/MS cytokinin analyses were again performed. In contrast to the previous work, methylthiol-derivatives were not detected in the seeds and *t*Z type cytokinins were present in the developing seeds, usually at higher levels than the iP type cytokinins. The transition to iP types previously reported [67] is not apparent in these cultivars, including in the cultivar in common [68]. 

These contrasting analyses potentially highlight the challenges of working with field-grown versus greenhouse material, and also of working with agronomic species that have been subjected to intense selection pressure through decades of breeding. 

##### Pea

It is clear that pea seeds develop considerably more rapidly than white lupin seeds, with the liquid endosperm in pea having disappeared by 10 to 12 DAA [11], which is in contrast to white lupin having extractable endospermic fluid at 46 DAA [13]. In pea, pod extension occurs concurrently with the syncytial phase, expansion of the endosperm, morphogenesis and embryo cell division in the seed. At the stage when the seed coat has peak sucrose levels, pod extension growth is completed and cotyledon expansion begins [65]. 

Three rapid phases of seed growth separated by two lag phases have been identified during development of pea seeds [105]. The first growth phase was confined to endosperm and seed coat and the second one was associated with the embryo, continuing until embryogenesis was completed. Up to this stage the embryo mainly grew by cell division. Another lag phase preceded the third growth period comprising the maturation phase, characterized by cell expansion of the cotyledons and storage product accumulation. Bioassay data indicated that peak cytokinin activity occurred during maximum volume of the endosperm, and during the two maxima in the growth rates of the whole seed and the embryo [109]. 

Across various stages of development and different pea tissues, LC-MS/MS analyses showed predominantly iPR and cZR and their nucleotides, with the ribosides most prominent in the young undissected fruit and cZR most prominent in the seed coat, relative to other cytokinins and tissues at later stages [63]. However, in other LC-MS/MS analyses [64], iPRMP was the most elevated form during the early to mid-developmental stage of cotyledon development in undissected pea seeds. 

In pea, a recent LC-MS/MS analysis of dissected pods and seeds [65] showed the total cytokinin content of the pod walls was relatively constant, with cZRMP the most significant form in ovules at 1 and 3 DAF, and in the pod wall. Relative to pod wall and cotyledons, the seed coat contained the greatest levels of cytokinin, predominantly as iPRMP with cZRMP also present. The iPRMP in the seed coat peaked at 14 days after fertilisation, which aligns with the main cytokinin previously detected in whole seeds [64]. Peak cytokinin in the seed coat coincided with the peak of sugar/starch in the seed coat and the beginning of cotyledon expansion. A decrease in total cytokinins was apparent in the cotyledons over time, with the exception of cZRMP at 20 DAF. Storage reserves were noted to increase markedly from 20 days after fertilisation, by which time cytokinin levels had decreased [65].

The very high levels of cytokinin detected in the endosperm of white lupin [12,13] were not detected in pea, possibly masked in pea by its much more rapid development and absorption of the liquid endosperm [11]. However, no attempt was made by [65] to trap endospermic fluid from the whole seeds. In contrast to the tight correlation between the peak of cytokinin in cereals soon after anthesis and cell division (see below), there was not a noticeable peak in cytokinin during the free nuclear phase within the first few days post fertilisation in the combined fruit tissues, although all four free bases, their ribosides and nucleotides were detected, with *c*ZRMP being the most noticeable in young fruits at both 1 and 3 DAP [65]. Interestingly, *PsIPT* gene expression decreased in pea ovules immediately after fertilisation [110], implicating maternal supplied cytokinin as the source of cytokinin in the newly developing pea fruit, as suggested for white lupin [13]. 

#### 3.1.3. Summary

In summary, there is no clear evidence for enhanced cytokinin activity during the earliest stages of fruit development in legumes, indicating a dependence on xylem/phloem supplies from the parent plant. Exogenous application also indicates that cytokinins are limiting for pod set in legumes (see references in [68,110]). However, following pod set, removal of seeds stops pod growth. Growth of the pod wall, which was most rapid during the first week after full bloom, involved cell division followed by cell enlargement and depended on the fertilized ovules being allowed to continue their development. Pod wall growth stops if seeds are killed or removed [11,111], indicating a close interaction between seeds and pod wall. Here, predominantly, gibberellin and auxin have been implicated [111].

No analyses of legumes covered both fully dissected seeds (seed coat, endosperm, embryo, cotyledons and embryo axis) and sampling over close time frames. The extraordinarily high cytokinin levels in the endosperm of white lupin certainly warrants further investigation to determine the origin of the cytokinin, be it filial or translocated from maternal tissues within the pod or seed.

The enhanced cytokinin levels in seed coats of several legume species places particular emphasis on this maternal tissue as a significant point of control. For pea, both the endogenous data and the gene expression data [65] indicate that the seed coat is a likely site of cytokinin biosynthesis. Interestingly, in double transgenics of pea simultaneously expressing an amino acid and a sugar transporter, a major effect was to prolong the peak of iPRMP accumulation in the seed coat [65]. The extended elevation of cytokinin in the seed coat of the transgenic pea was matched by enhanced cell wall invertase (*CWIN*) expression in the seed coat and related to increased sucrose in the cotyledon. The transition from the morphogenesis to maturation and storage activities has been associated with a decrease in invertase expression and activity [9,112]. In the double transgenic lines, the expression of *CWIN* continued into the maturation phase and appeared to disturb the clear transition from morphogenesis to storage activities [105,113], supporting the contention that the higher yield of the transgenic peas might be due to increased sink strength enabling the plant to fill more seeds [65]. Notably, transgenic *Vicia narbonensis* seeds overexpressing a *V. faba AAP1* also showed significantly greater cytokinin content, particularly 15 DAF, with the suggestion that increased seed yield was, at least in part, due to the extended period of seed filling [114]. 

Cell wall invertases and cytokinins are considered important regulators of sink strength (e.g., [115]). Consistently, *CWINs* are highly expressed in post phloem regions with no plasmodesmatal connection, including the interface between seed maternal and filial tissues (reviewed in [116]). For example, strong CWIN activity was detected in the innermost cell layers of the *Vicia faba* seed coat and the outermost cell layers of maize filial tissues—the basal endosperm transfer cells (cited in [116]). The potential interaction of cytokinin with CWIN in the seed coat of the double transgenics was related to increased sucrose in the cotyledon, and an overall increase in seed protein content and seed number at maturity [61].

However, the peak of iPRMP in the control seed coats occurred when cell division in the embryo would have ceased, as this is essentially finished by the time storage products accumulate. There may be no further requirement for cytokinin in the embryo itself, except as a source during germination. Notably, the cytokinins present in seed nearing maturity in yellow lupin were resistant to CKX (DZR and DZROG) [61]. 

### 3.2. Cereals

#### 3.2.1. Source of Cytokinins in Cereals

Exudate from young stems of wheat contained bioassay-active, cytokinin-like substances that could move through the stems to the ears [43]. A positive correlation between cytokinin content in roots of rice and those in grains was shown [117] and, furthermore, when kinetin was applied to rice during early endosperm development, both endosperm cell number and endosperm/grain weight increased, and more so in the generally smaller inferior spikelets than in the superior spikelets. This effect was more pronounced when kinetin was supplied to roots than when it was sprayed onto leaves and panicles, with the conclusion that the roots were the major source of cytokinin for the grain [117]. Relatively few other experiments investigating translocation of cytokinin have been undertaken in cereals in a fashion similar to those undertaken in legumes. Several cytokinins from root exudate of rice have been identified by GC-SIM, including *t*Z, *t*ZR, *c*ZR and *t*ZRMP [118], and additionally *c*Z, *c*ZOG and *c*ZROG [42]. Multiple cytokinin forms have also been detected in wheat and oat xylem sap from seedlings and quantified by RIA following exhaustive separation procedures: *t*Z, DZ, *t*ZR, DZR, their *O*-glucosides and nucleotides, *c*Z and *c*ZR were all detected [119,120], incidentally confirming the presence of *cis*-isomers as endogenous cytokinins and not artifacts of isolation procedures [72]. 

#### 3.2.2. Sites of Cytokinin Accumulation in Cereal Grains

When comparing cytokinin content with stage of development, it is important to be aware that development in wheat grains, barley and rice post pollination is considerably more rapid than that, for example, in maize. Moreover, it is important to be aware that there are waves of development within an ear (most advanced in the middle spikelets of a wheat ear) and within grains within a spikelet [44], and different rates of development in superior and inferior grains from rice panicles [117]. Analyses of whole spikes or indeed spikelets will likely mask rapid changes. 

The development of cereal endosperms progresses through coenocytic nuclear division (0–2 days after pollination (DAP) and cellularisation (3–5 DAP), followed by mitotic cell divisions and aleurone, transfer cells and starchy endosperm differentiation. Storage product accumulation begins and, subsequently, the large central starchy endosperm cells begin to undergo programmed cell death, a process that progresses from the centre of the endosperm toward the crown and then the base, finally affecting the entire tissue except the aleurone cells [9]. 

In cereals, there is a general consensus that the developmental events that precede the rapid accumulation of storage material are crucial for establishing the capacity for endosperm growth and therefore yield, and summaries of early work [121,122,123] concluded that endosperm cell proliferation is accompanied by transient but marked increases in kernel cytokinin content in barley, maize, rice and wheat.

Below is a more detailed look at individual components of the transient increase, the timing of that increase with respect to the stage of development, and the expression of *IPT* gene family members (GFMs) in view of the statement by Radchuk et al. (2023) [102]: *“*Whereas some authors have suggested that cytokinin is synthesized early in the developing seed (discussed in Jameson and Song 2016), the transcription of genes encoding the components of cytokinin synthesis in cereals does not correlate well with the sites of cytokinin accumulation during the grain filling stage (Mrízová et al., 2013; Powell et al., 2013; Hluska et al., 2016)”.

##### Wheat

Within the wheat ovule, changes occur very rapidly following pollination. Free nuclear divisions occur within a few hours of flowering, and cellularisation of the free nuclear endosperm begins at 1 to 2 DAA and is complete within three to four days. Mitotic cell divisions subsequently occur and have decreased substantially by 9 DAA and ceased by about 12 to 14 DAA. Generally, by 9 DAA, embryo differentiation is nearly complete. Soft dough stage is from 14 to 21 DAA, and hard dough from 21 to 31 DAA (e.g., [46,124]). Final grain size appears to be closely related to the number of endosperm cells formed after anthesis [125]. 

The pattern of cytokinin activity in developing wheat grains shown by bioassay data [44] and that from wheat spikelets by immunoaffinity purification/HPLC/RIA [126] is remarkably similar, especially when [126] is recalculated on a pmol/g FW basis (ca. 280 pmol/g FW; Z >> ZR). Both show a sharp increase in cytokinin immediately after anthesis, peaking at 2 to 4 DAA and reducing rapidly to near baseline by 9 DAA. 

The levels of six cytokinins were determined in grains from 0 to 25 DAA, with extracts passed through a C_18_ column and then separated by HPLC prior to ELISA [124]. Whole kernel hormone levels declined from 3 DAA [Z (30), ZR (1.8), other cytokinins < 0.6 nmol/g dry mass] to low levels by 9 DAA and remained low thereafter. At 13 DAA, Z content in embryos was 0.9 nmol/g DM, which then decreased to 0.3 nmol/g DM by 25 DAA. Total iP and iPR levels were low between 0 and 6 DAA but, in contrast to other cytokinins, tended to increase slightly between 13 and 25 DAA [124].

The cytokinin content of five wheat cultivars was analysed by LC-MS/MS [46]. In general, in the cultivars grown in the field in Canada, *t*Z and *c*Z were at relatively similar levels at 1 DAA and 4 DAA. Two of the high-yield cultivars, HY124 and SW222, accumulated more *t*Z than did the low-yield cultivars, especially at 4 DAA, although the *t*Z and *c*Z levels were similar [46]. This is in contrast to the cytokinin levels analysed by LC-MS/MS in wheat cv. Torch grown in the field in New Zealand, where *t*Z levels greatly exceeded those of *c*Z at 4 DAA [47]. In agreement with [46], *c*ZOG and *c*ZROG exceeded the level of their *trans* equivalents, and both papers reported elevated levels of *t*Z9G and low levels of iP types at 4 DAA, with total cytokinin levels peaking around 1.1 nmol/ g FW [46] and 1.6 nmol/ g FW [47]. 

In contrast to the statement by Radchuk et al. [102], the expression of *TaIPT* GFMs [85,127] is co-incident with the increase in *t*Z, peaking between 2 and 4 DAA in developing wheat grains [44,46,47,85] and spikelets [126]. Further, the time-series transcriptome profile of developing wheat grains at 0, 2, 4, 6, 8 and 10 DAP [127] showed peak expression of an *IPT* and a *LOG* GFM at 2–4 DAP, and enrichment of B-type RRs between 2–6 DAP [127]. The peak of *TaIPT* expression shown in [46] is between 4 and 7 DAA. Notwithstanding this difference, these data all correspond with the transition to and/or phase of rapid mitotic divisions in the endosperm and not the grain filling phase. Notably, growth immediately following anthesis takes place mainly in the pericarp. While starch has been shown to accumulate in the pericarp soon after anthesis, starch accumulation in the endosperm may not begin until the pericarp is fully grown, at about 17 DAF [128], which is in agreement with data showing the linear phase of starch accumulation in whole grains beginning around 17 DAA, coinciding with the peak of grain sucrose levels [129].

Cytokinins susceptible to β-glucosidase treatment accumulated in wheat tissue pre-and post-fertilisation [44], as did transcripts for *cis*-*O*-glucosyl transferases (*cis-OGTs*), but not transcripts for the *trans*-*OGTs* [85]. *TaCKX* GFMs were also elevated. Noteworthy also is the elevated expression of cytokinin β-glucosidases peaking between 4 and 7 DAA [85], which could lead to release of active cytokinin.

Daily analyses of both cytokinin content and gene expression in pericarp, endosperm and embryo are clearly warranted to determine the location and timing of these key factors owing to the rapid development of the wheat grain. More attention should be paid to the early development of the pericarp in wheat, considering the noted accumulation of cytokinin in the seed coat of legumes.

##### Rice

During the early phase of rice grain development, the syncytial nuclei are evenly arranged in the peripheral cytoplasm surrounding a large central vacuole [130]. At three DAF, nuclear divisions in the peripheral syncytium ceased and by 4 DAF cellularisation of the endosperm had occurred. The timing of cellularisation of the syncytium is considered a determinant of seed yield, as it determines the number of cells available for filling during seed storage ([130] and references therein). When the number of endosperm cells was calculated based on the number of nuclei present, the data indicated that the cell division rate peaked at 6 DAP, and the maximum cell number was attained between 10–12 DAP for superior spikelets, and at 12–14 and 22–24 DAP, respectively, for inferior spikelets [117,131].

The most comprehensive analysis of cytokinins in rice grains saw multiple cytokinins identified and quantified by GC-MS and GC-SIM, with the greatest amounts of cytokinins recorded at early developmental stages, namely either heading, anthesis or milk stage [56]. While *c*Z and *c*ZR were high at heading and decreased rapidly after anthesis in grains, all other detected cytokinins accumulated after heading, either peaking at anthesis (*t*Z, *t*ZR, Z9G, *c*ZOG, *c*ZRMP, *t*ZRMP) or at milk stage (*c*ZROG and *t*ZROG). Only *c*ZOG and *c*ZROG were detected at maturity, the content of most cytokinins having declined rapidly to low levels at dough stage. The authors stated that the ‘anthesis’ stage was where cell division in the embryo and endosperm was culminating and turnover of bioactive cytokinins was apparent [56]. *cis*-Isomers were always more abundant than *trans*-isomers and, because of the large changes in the contents of the *cis-*isomers in the rice grain, the authors suggested that not only were the *cis-*isomers being synthesized and metabolized, but also that the source of the *cis-*isomers might not be restricted to tRNA turnover and might involve de novo synthesis [56]. As rice has OGTs with specific activity against *c*Z types [50], the presence of *cis*-specific OGTs may permit the accumulation of *O*-glucosylated *cis*-isomers, which are resistant to CKX [31]. Their turnover would release *cis*-forms, which are susceptible to CKX. High levels of *cis*-OGTs were also reported in wheat, both pre-and post-anthesis [85]. The early peaks of biologically active cytokinins in rice [56] were not shown in Table 2 of Chen et al. (2020) [123].

While there is little cross referencing to previous work at the same site, both groups showed that cell number and cell division rate in rice endosperms varied among genotypes and with position of spikelets within a panicle [117,131]. A total of eight genotypes were assessed, with samples from superior and inferior spikelets purified by passage through C_18_ SepPak cartridges prior to ELISA. Z + ZR contents in the endosperm correlated positively with the rate of endosperm cell division and the total number of endosperm cells. Genotypes with a faster rate of endosperm cell division also had more Z + ZR in the roots during early endosperm development, and the root content paralleled that in the grains. When kinetin was applied to shoots or roots, an increase in endosperm cell number in inferior but not superior spikelets occurred after kinetin was applied to roots [117,131] and to shoots [131], leading to differently worded conclusions: that root-derived Z and ZR play a pivotal role in regulating cell division activity in the endosperm [117], and that Z and ZR both in grains and roots play important roles in regulating post-anthesis development of spikelets in rice [131].

More recently, publicly available databases were used to analyse the expression patterns of *OsIPT*, *CKX* and *LOG* GFMs in ovules and in grains at 2, 6, 9 and 16 DAF [15]. The study showed that *OsIPT4*, *OsIPT5* and *OsIPT7* were expressed in endosperm but not the embryo, up to 9 DAF. Interestingly, LOG GFMs were almost constitutively expressed in the embryo [15]. Using LC-MS/MS and analysing ovaries before fertilisation, seed at 3 DAF and endosperm at 6, 9 and 16 DAF, both ZR >> Z were detected but only at 3 and 6 DAF, which is consistent with the expression patterns of the *OsIPT* GFMs. Using a starchy endosperm-specific promoter to drive *OsIPT3*, the authors reported increased Z levels at 8 DAF, and increased length and suppressed width of seeds, resulting in slender grains with a greater grain weight but reduced quality [15]. They concluded that cytokinins have more crucial roles in endosperm than in embryo, and that cytokinins were produced at the early stage of endosperm development to initiate and maintain cell division or syncytium formation.

Therefore, in rice, cytokinin accumulation and *OsIPT* expression appear coincident in the endosperm and both had declined before the major grain filling period. The cytokinins present at maturity were inactivated storage forms, i.e., *c*ZOG and *c*ZROG [56].

##### Maize

Development of the maize kernel involves more obvious cellular differentiation than observed for other cereals. During the lag phase of growth, from 0 DAP to as late as 15+ DAP, kernel dry weight gain is minimal as the endosperm and embryo develop, differentiate, and increase in size [132,133]. During this lag phase, the endosperm first undergoes free nuclear development creating a multinucleate coenocyte composed of several hundred nuclei. Subsequent wall formation yields a completely cellular endosperm such that by 4 d after pollination (DAP) the endosperm is completely cellularised. This is followed by the mitotic phase, an endosperm-wide proliferation of cells, which is essentially complete by 10 to 12 DAP. By the end of the lag phase, mitotic activity becomes restricted to the peripheral layers of the endosperm [132,133].

Coincident with cell proliferation, four major cell types with specific functions differentiate within the endosperm: the aleurone, basal endosperm transfer layer (BETL), embryo surrounding region (ESR), and starchy endosperm (SE). An additional 3–4 cell types develop later. By the end of the lag phase, the endosperm accounts for about 60% of the kernel volume. During this phase, the embryo differentiates. Moreover, during the early lag phase, the maternal nucellus tissue at first expands and accounts for a significant portion of the kernel, but by about 12 DAP it degenerates and remains only as the nucellar membrane. The exterior pericarp also expands and starts to develop thickened walls [132,133]. During the linear or grain-filling phase of seed development (from about 12–40 DAP), as endosperm cell proliferation slows, there is rapid water and weight gain as the SE cells expand with deposition of storage compounds and multiple rounds of endoreduplication. SE cells accumulate carbohydrates in the form of starch and seed storage proteins accumulate in protein bodies. Fully differentiated SE cells begin the process of programmed cell death (PCD). In the linear phase, the embryo continues growth and development and the pericarp cells begin to die. The final maturation stage of maize kernel development involves PCD of all endosperm cells except the aleurone, final development of the embryo and kernel desiccation and quiescence [132,133].

The first 10 to 12 DAP is a critical period during kernel development in maize as the capacity of the endosperm to accumulate dry matter (kernel sink capacity) is established during this period and has been shown to be a function of the number of endosperm cells formed (i.e., cell division) and the number of starch granules formed (i.e., amyloplast biogenesis) (reviewed in [134]).

Again, early work shows peaks in cytokinin levels (ZR usually > Z) between 4 and 12 DAP (see references in [123,134]), coincident with the phase of high endosperm cell division activity. Following anion + cation exchange and immunoaffinity purification followed by HPLC-diode array quantification, the ZR and Z levels of in vitro control and field-grown kernels peaked at approximately 9 to 12 DAP, with ZR > Z peaking in low ug/g FW levels [134]. These researchers also recommend daily sampling as necessary to detect the peak of cytokinin. Using cation exchange and immunoaffinity purification, followed by HPLC and radioimmunoassay of fractions, daily sampling showed ZR and Z at low levels until 6 DAP and peaking at 9 DAP (ZR > Z). The authors commented that endosperm cell division was well advanced at this point [126].

Generally, little attention has been paid to *cis* derivatives, *O*-glucosides or nucleotides, all of which have been identified in maize kernels. LC-MS analyses revealed that *cis*-zeatin was present in roots, stems, leaves, unfertilized cobs and kernels, along with its riboside and nucleotide [32]. Moreover, the *O*-glucosides of *cis*-isomers were found in roots, young cobs and kernels, which aligns with the expression of *cisZOG1* and *cisZOG2* in maize tissues. However, they also noted that *cis*-isomers were more prevalent in roots, stems, and leaves, whereas *trans*-isomers were more abundant in the kernels. The levels of DZ and iP types were relatively low [32].

The research carried out by Brugière et al. [135,136] and Rijavec et al. [115,137], and described in some detail below, used immunoaffinity purification and HPLC-diode array quantification of cytokinins, which excludes *O*-glucosides from analysis.

Strong evidence for the coincidence of *IPT* gene expression and the accumulation of cytokinin in regions of the developing maize kernels can be seen when gene expression and endogenous cytokinin content in upper and lower parts of developing kernels are compared [135,136]. *ZmIPT2* was transiently expressed during kernel development and reached a maximum expression at 10 DAP. This expression pattern coincided with the peak in rate of cell division (referring to [138]) and the peak of CK accumulation in the endosperm during kernel development [135]. Importantly, in the divided kernel, the cytokinin levels in the pedicel/placental chalazal/basal endosperm region were two to three times higher than in the rest of the grain [135], and the expression of *ZmIPT2* in this tissue was greater than in the rest of the grain and closely paralleled the cytokinin level differences between the two parts of the grain [136].

Using Northerns, the expression pattern of *ZmIPT2* from 0 to 25 DAP was further investigated in kernels dissected into the pedicel/placental chalazal/transfer cell region, nucellus, starchy endosperm/embryo sac, starchy endosperm, embryo and pericarp [136]. The results confirmed that *ZmIPT2* transcript levels in the pedicel/placental chalazal/transfer cell region were more abundant than in the rest of the kernel. This is especially the case at 15, 20 and 25 DAP. Strong *ZmIPT2* expression was also observed in the starchy endosperm/embryo sample at 10 DAP. At 10 DAP, embryo cells are difficult to separate from the starchy endosperm but represented a very small percentage of the cells in this tissue. Expression was absent from the nucellus and was low in the developing embryo at 15, 20 and 25 DAP compared to pedicel/placental chalazal/transfer cell region and the starchy endosperm/embryo sample at 10 DAP. In the pericarp, expression was just detectable at 5 and 10 DAP but undetectable at later stages [136]. These results indicate that the maternal pedicel/placental chalazal and adjacent filial transfer cell region is most likely a strong site of cytokinin biosynthesis. The presence of *ZmIPT2* transcripts in the endosperm/embryo sample at 10 DAP and in the developing embryo at 15, 20 and 25 DAP was observed at times when cell division was most active in these tissues [136].

Further investigation using immunolocalisation of ZmIPT2 at 8 DAP indicated that the protein was below the limit of detection in the maternal pedicel, but was present at a high concentration in the filial endosperm transfer cell layer and at lower concentration in the starchy endosperm, with the conclusion that ZmIPT2 most likely plays a role in endosperm cell division during the peak of mitotic activity in the endosperm [136]. However, the researchers also suggested that the presence of the ZmIPT2 protein in the endosperm transfer cell layer both during the peak cell division period and at later stages of kernel development strongly suggested that cytokinin could also play an additional role in kernel sink strength [136]. As mentioned earlier, strong CWIN activity has been detected in the outermost cell layers of maize filial tissues, the basal endosperm transfer cells (cited in [116]) and cross talk between cytokinin and CWIN is well recognised in the provision of hexoses to facilitate cell division and cell expansion [115,139].

The specific question of whether the “huge amount of cytokinins detected in maize caryopsis is a consequence of their transport from the maternal tissues to the filial tissues or de novo biosynthesis of cytokinins within the seed itself” was addressed by Rijavec et al. [137]. Much of this research supports that published by Brugière et al. [136]. Caryopses comprising maternal pedicel, nucellar and filial tissues showed a 60-fold increase in cytokinin levels relative to the unpollinated ovules at 0 and 6 DAP. The two main cytokinins in caryopses at 6 and 8 DAP were *t*Z and *t*ZR. The greatest levels were in the more basal section, and detectable levels of *t*Z, *t*ZR and *t*Z9G remained at 12, 16 and 20 DAP [115]. However, their measurements of cell divisions and cytokinins in the corresponding caryopsis did not show strong agreement. They noted that the cytokinin peak appeared in the early days after pollination, well before the peak of cell divisions [115].

At 12 DAP, endosperm and embryo were able to be separated and the two tissues showed very different cytokinin content: ZR was the most abundant cytokinin in the endosperm but was only detected in trace levels in the embryo, whereas Z9G was predominant in the embryo [137]. Cytokinin levels in the embryo were always lower than those in the endosperm. *O*-glucosides were not monitored, and *cis*-isomers were not mentioned.

A strong cytokinin immuno-signal was detected in specific cell types in the pedicel, endosperm and embryo, but little was seen in the nucellar region [137]. At 8 DAP, the immuno-signal for cytokinins was detectable in the BETL region and a relatively strong cytokinin signal was present throughout the endosperm from 8 to 12 DAP. At 8 DAP there was a clear delineation of the embryo surrounding region (ESR) from the rest of the endosperm by a very strong cytokinin signal. After 12 DAP, most of the endosperm immuno-signal was localized to the outer layers of the endosperm, particularly the aleurone with the labelling overlapping with the cessation of cell divisions in the central endosperm and their continuation in the peripheral cell layers, away from the embryo until late developmental stages [137].

In situ hybridisation analyses of *ZmIPT1* at 7 and 14 DAP indicated that the location of cytokinin biosynthesis was spatially restricted to cells surrounding the vascular tissue consistent with immunolocalisation of cytokinin in the pedicel region of developing maize caryopsis [137]. However, *ZmIPT1* is a *tRNA-IPT* and was shown to be highly and constitutively expressed in all tissues [140]. Significantly, turnover of tRNA would be contributing to the pool of *cis*-isomers (and not to the pool of *trans*-isomers), compounds on which Rijavec et al. [115,137] did not report. Notably, the *ZmCNGT* transcript was also readily localized in the pericarp, scutellum, and pedicel region, but Z9G accumulated only in the embryo [137].

The conclusions from this work [137] were that the high levels of cytokinin in the filial tissue coincided with the early phase of development known to be associated with DNA replication and cell division in the endosperm, but that the caryopsis cytokinins may have other physiological roles. The authors suggest that the strong immunosignal in the pedicel indicates high cytokinin levels, which they proposed may have triggered developmental programmed cell death in the pedicel [137].

The role of cytokinins in early embryogenesis and endosperm development in maize was investigated using the promoter TCSv2 (two component system, cytokinin-responsive promoter version #2) to drive the nuclear-localized RFP tdTomato reporter in order to visualise where cytokinin might be active [141]. The study showed that TCSv2 activity is apparent in the endosperm before cellularisation at 3 DAP. By 3 DAP, strong signals were detected in the nucellus and integumental cells at the micropylar area, but not inside the embryo. By 6 DAP, TCSv2 activities were apparent in the endosperm chalazal area and the BETL at the pro-embryo stage, the early transition stage (7 DAP) and towards the late transition stage (8 DAP). From 8 DAP, TCSv2 activity was increasingly detected in parts of the embryo [141]. In support of these data, a transcriptome analysis of extracted pure maize endosperm showed cytokinin-activated signalling pathway genes were particularly enriched in the endosperm at 6 DAP [142].

The most comprehensive LC-MS/MS cytokinin analysis across a range of developmental stages in maize showed that the ratio of *t*Z type to iP type cytokinins was strongly in favour of *t*Z types in ovules and developing kernels [31], as indicated in a number of studies. As noted in wheat and rice before pollination [44,56,85], the maize ovule also appears to be a place of active cytokinin metabolism [31]. However, in maize there was also cytokinin biosynthesis occurring in the ovule because the primary biosynthetic products, *t*ZRMP and iPRMP, were the most abundant cytokinin derivatives there, with cZROG also accumulating [31]. Much earlier, it had been noted that unfertilised ears of maize had high levels of bioassay-active cytokinins [21]. In developing kernels, the greatest accumulation of *t*Z and *t*ZR was detected between 12 and 20 DAP. *t*Z- and *t*ZR *O*-glucosides accumulated predominantly at 12 and 20 DAP in tissues where high *t*Z and *t*ZR content was detected. In contrast to *t*Z, *c*Z occurred predominantly as the *O*-glucoside. An interesting comment was that, in contrast to the changing *t*Z and *t*ZR levels, there were consistently low levels of cZ and high levels of cZROG over the course of development (implicating removal to non-active forms), with the exception that they had all but disappeared by 35 DAP [31]. The high cytokinin was detected in kernels up to 20 DAP, where maximal cell division occurs, and had a profile similar to that shown earlier [136]. Interestingly, both studies mention accumulation of iP type cytokinins at 20 DAP, which may be relevant considering the sensitivity of a maize receptor to iP [78]. Accumulation of *O*-glucosides was noted—especially as *cis*-isomers—and, as mentioned earlier, this allows *c*Z to accumulate due to the action of *cis*-specific OGTs, as *c*ZOG is protected against degradation by CKX [31] but can be turned over to release *c*Z.

In contrast to the marked decrease of *t*Z and *c*Z types by 35 DAP, DZ types had accumulated in the desiccating kernels by this time [31]. Accumulation of DZ types in dry maize seed was also shown [87], and particularly DZR and DZROG [30]. This may be of importance because these cytokinins are resistant to side chain cleavage by CKX and, therefore, may be conserved to contribute to the germination process.

Therefore, in maize, *ZmIPT2* expression and immunolocalisation, elevated cytokinin levels and activation of cytokinin signalling all point to biosynthesis and activity of cytokinin in the endosperm and in the BETL region, correlating with cell division, the establishment of sink tissues and the growth of the embryo.

##### Barley

During the early development of the barley grain, cellularisation of the syncytium was initiated at 3–4 DAF [11,143]. Endosperm transfer cells were the first to differentiate around 3 DAF, while the remaining peripheral layer is still retained in the syncytial stage. Around 6 to 7 DAF, after repeated mitotic divisions, cells of the aleurone layer can be identified. Starchy endosperm cells are derived from the inner daughter cells of the aleurone layer and subsequently fill the centre of the endosperm [11,143]. A transition from metabolic activities to storage occurs between 10 and 12 DAP [144].

The cytokinin content of field-grown, high- and low-yielding barley lines was examined using LC-MS/MS [53]. The highest concentrations of *cZ* forms were associated with floret setting and those of *t*Z forms were associated with the kernel filling stages, with the suggestion that *c*Z corresponded to the initiation of kernel elongation and was involved in the process of determining the number of cells available for starch storage [53]. Unlike other analyses of cereal kernel development, which show cytokinin levels decreasing to near baseline before the peak period of starch accumulation in the kernels, barley cytokinins increased to maximal peaks and, in particular, to very high concentrations of *t*Z (23,909–48,294 pmol/g FW—i.e., 48 nmol/g FW) at the late milk/soft dough stage, 10–12 DAP. The recent paper by Radchuk et al. (2023) [102] cites Powell et al. (2013) [53], stating that “*t*ZR represents the bulk of cytokinin molecules in the developing barley grain”. In fact, the *t*ZR levels are always less than the *t*Z levels and are 10-fold less at the peak (2752–4384 pmol/g FW) than the *t*Z levels [53].

In contrast, at the earliest stages of barley grain development, when an excess of *cis*-isomers compared with *trans*-isomers was indicated [53], other LC-MS/MS data show highly elevated *t*Z levels in barley grains at 4 DAP, followed by a sharp decrease in total cytokinins. While low levels of some *cis* derivatives are identified [51], there is no mention of *c*Z itself. Moreover, there is no evidence in the samples from Germany [51], taken at 8, 10, 12 and 14 DAP, of the high *t*Z levels shown by [53] at 10–12 DAP. Interestingly, both sets of cytokinin analyses appear to have been performed in the Emery lab. In barley cv. Quench grown in New Zealand, analysed at a single time point by LC-MS/MS, *t*Z levels were considerably greater than those of *c*Z at 4 DAA [47], which aligns not only with the data from Faix et al. (2012) [51] but also with that from wheat [47]. In data covering wide time frames, at no stage of development did cytokinin levels in wheat reach those reported for barley [53], nor is there any suggestion, by bioassay or chemico-physical means, of elevated *t*Z in wheat or rice after the early peak of activity, although there is mention of the detection of the iP and iPR in mature wheat grains [46,124]. Analyses of whole barley spikes at one timeframe [54] when the first grains have reached half of their final size do not help to resolve these differences. Notably, *c*Z was not detected [54].

In the above papers, grains were not separated into different tissue types. Interestingly, dissected barley grains showed enhanced transcript abundance particularly for *HvIPT5* in the husk, spikes and endosperm, and for *tRNA-IPT1* in the embryo, leading to the conclusion that de novo cytokinin production is likely reduced in embryos where no *HvIPT5* transcript was detected [145]. The stage of development of the dissected grains was not specified. In a different analysis, barley *tRNA-IPT1* was shown to be highly expressed in barley spikes at 7 DAP relative to other *HvIPTs* [146]. The *tRNA-IPT1* would only be contributing *cis*-cytokinin derivatives.

Using barley transcriptome data from Monat et al. [147], Radchuk et al. [102] showed elevated levels of cytokinin signal transduction pathway genes (*AHPs*) in 5- and 15-day-old caryopses, and suppression of response regulator A (*RRA*) GFMs in both 5- and 15-day-old caryopses, both indicative of active cytokinin signalling. No *HvAHP* transcription was detected in 4-day-old embryos, while there was no RRA expression data shown for embryos [102]. It is a pity that *IPT*, *LOG*, *CKX* or *CGT* GFMs were not abstracted from the Monat data set [147], particularly as it has been suggested, albeit in early developing rice panicles, that the variation of expression during panicle development is greater among genes encoding proteins involved in cytokinin biosynthesis (*IPT, LOG*), degradation (*CKX*) and negative regulators (*RRAs*) of the pathway than for the genes in the primary response pathway [148].

Irrespective of evidence for cytokinin biosynthesis in endosperm tissues of various cereals and the fact that no other cereals, including other analyses of barley, show elevated levels of cytokinin during grain fill, Radchuck et al. (2023) [102] base their paper on the high levels of *t*Z and *t*ZR reported in barley grains by Powell et al. (2013) [53] and state that “achieving the high levels of *t*Z and *t*ZR present in barley grains (Powell et al., 2013) must depend on their supply from maternal tissues”.

RNA-seq performed on 6- and 12-day-old grains showed that four *HvAHP* GFMs were transcribed during early development, while another six were transcribed during grain filling, in agreement with the transcriptome data. It was shown that the later expression during grain filling coincided with expression of *HvSWEET11b*. The only RRA (RRA2) detected showed modest expression in developing grains [102].

In the same paper, HvSWEET11b was shown to transport cytokinins in the *Xenopus* assay, but with *t*Z transported more efficiently than iPR > *t*ZR. In the same assay, AtSWEET15 transported only *t*Z and not the ribosides. Apart from SWEET11b, no cytokinin transporters (either ABC14 or PUP14) were transcribed during grain filling (based on analysis of the database [147]), with the authors suggesting that other known cytokinin transporters cannot functionally replace HvSWEET11b in terms of cytokinin transport during grain filling [102].

Based on an LC–MS metabolomics platform, whole grains sampled at the start of the grain filling stage from HvSWEET11b-repressed plants contained higher levels of *t*ZR than WT grains [102]. However, only relative levels are provided by the platform and absolute levels (e.g., in pmol/g FW) for comparative purposes with published papers are not provided. Furthermore, the authors used a Fourier transform infrared (FTIR) analysis technique in an attempt to quantify *t*ZR in cross sections of transgenic and WT grains and to examine the spatial distribution of *t*ZR. That this technique could identify crystals of *t*ZR seems highly unlikely, given the generally low levels of cytokinins, and it is likely that the spectrum is filled with cross-talk from other structurally related molecules, resulting in a gross overestimation of *t*ZR (Charles Hocart, pers. comm.) in the maternal vascular bundle, surrounding pericarp and nucellar projection of transgenic grains and WT. Given that *t*Z is reported to be at a 10 times greater level [53], and to be transported more efficiently than *t*ZR, it seems relevant that this cytokinin was reported as being below the detection limits of the FTIR technique [102].

Furthermore, Radchuk et al. [53] state that “Both sugars and cytokinins are brought to the maternal–filial boundary through the maternal plant’s vascular system”. In contrast, maize kernels grown in vitro with a source of auxin and in a high sugar environment developed normally [139], indicating that kernel development was not reliant on a source of cytokinin via the vascular system from the parent plant [115]. However, if indeed the cytokinin is being produced by maternal seed tissues and high levels of *t*ZR are being transported to the interface between maternal and filial tissues and released into the apoplast by SWEET11b, one might look for activity of CPN1 in the apoplast of the filial tissue (in this case the endosperm where the putative high *t*Z and *t*ZR levels reside) to convert the *t*ZR into active *t*Z [34]. Then, receptors at the plasma membrane of the endosperm could be activated, precluding the need for PUPs to transport the cytokinin into the cytosol of the filial tissues.

#### 3.2.3. Summary

In summary, it is clear from multiple analyses in various grains that cytokinins accumulate rapidly during early grain development, with the cytokinin levels in maize somewhat attenuated compared with those in wheat and rice, potentially reflecting the larger size and slower development of the embryo and other differentiated structures. The correlation between gene expression, cytokinin accumulation and cell division in cereals (with the exception of [53] and [102]) point to a key role for cytokinins in establishment of the sink for storage product accumulation. However, in light of the accumulation of cytokinins in the seed coat of legumes, it may well be relevant to assess more closely the exact location of the early peaks of cytokinin in cereals and whether these could be in the seed coat equivalent of cereal grains.

## 4. Where to from Here?

Early biochemical analyses pointed to a role for the cytokinins in seed development, and comprehensive mass spectroscopic analyses show that a complex of cytokinins exists in all seeds examined so far. Both forward and reverse genetics approaches have confirmed a role for the cytokinins as a determinant of yield (refer [1,123]). Correlative analyses support this (e.g., [5,46,48,67,117,131]). Transcriptomics is adding to the picture (e.g., [127]), as well as high-resolution analyses such as laser-assisted microdissection technology, which has enabled the isolation and high-resolution transcript analysis of early endosperm development in, for example, arabidopsis [149] and barley [11]. Additionally, as shown for wheat [89,123,150], rice [15] and barley [102], much valuable transcriptomic information is ‘hidden’ in publicly available databases, but these data are usually at the organ level. Consequently, high-resolution transcriptomics, in situ hybridisation to locate the sites of cytokinin biosynthetic GFMs and immunolocalisation of biologically active cytokinins are all required to pinpoint precise sites and stage(s) of development of cytokinin function.

### 4.1. The Cytokinin Complex

From a metabolite perspective, the wide variety of cytokinins present in both legume seeds and cereal grains poses a number of questions. Making the picture more challenging, there are clear differences in the cytokinin types, not only between monocots and dicots and species within these, but there also appear to be some distinct differences between cultivars of the same species, for example in soybean [51,53,67], wheat [46,47] and barley [47,51,53]. These may be due to different analytical techniques or indeed may be real and a consequence of breeding both within and between countries [151], all leading to a somewhat challenging picture.

Is the accumulation of *cis*-cytokinin isomers nothing more than the conjugation of degradation products of tRNA turnover that occurs in all organisms at a constant rate [31]? Does *c*Z actually have a physiological role [36,77]? Dihydro derivatives are clearly prominent cytokinins in both cereal and legume seeds, yet the pathway to these CKX-resistant cytokinins is as yet unknown [77]. As DZ is regarded as a biologically active cytokinin, more information could be informative, but will not be obtained when only a limited number of cytokinins is targeted.

Several groups have recorded increased levels of the non-hydroxylated iP types during maturation [31,46,67,124,136]. The importance of hydroxylated derivatives is clear [36,42], but the identification of receptors responsive to iP in maize [78] and rice [79] might indicate biological relevance in the maturing seed.

Whatever technical approach is used, at the very least daily analyses are needed from the time of pollination through to and including the storage phase at the tissue and cell level, along with interactions with other plant hormones which, in the interests of clarity and to do justice to the complexity of the cytokinins, have not been discussed in this review.

### 4.2. Seed Set and the Transition from Syncytial Endosperm to Cellularisation

Do *cis* derivatives have a functional role in pod and seed set [13,53]? Are cytokinins involved in the syncytial divisions and/or the mitotic divisions occurring subsequently? Indeed, careful analysis of maize seed during coenocyte and cellularisation showed enrichment of auxin-activated signalling pathway genes in the coenocyte and enrichment of basipetal auxin transport genes during cellularisation [143]. In contrast, cytokinin-activated signalling pathway genes were only modestly enriched in the coenocyte, and somewhat more enriched during cellularisation [143]. The observation that overproduction of auxin prevents endosperm cellularisation in arabidopsis suggests that auxin levels have to decrease below a certain threshold in order for the endosperm to cellularise [127]. Likewise, increased cytokinin signalling in barley could induce a shift in the hormonal balances towards cytokinin that triggers the start of cellularisation in the syncytial region [11]. As the coenocytic phase and cellularisation prior to differentiation are so short, closer inspection and careful analysis of both cytokinin and auxin during these phases is clearly warranted. Indeed, Zhang et al. (2023) [152] recently suggest that the transcription factor TaMADS-GS repressed expression of *TaCKX* and stabilised cytokinin signalling during endosperm cellularisation in wheat. Knockout of TaMADS-GS led to delayed endosperm cellularisation [153], supporting a specific role for cytokinins in the initiation of cellularisation.

### 4.3. Morphogenesis and the Transition to Storage Functions

In the seeds under discussion, a critical factor is the relatively delayed growth of the structure to be nourished (the embryo) and the relatively precocious development of the nutritive material (the filial endosperm) at the expense of the maternal nucellus [107]. In angiosperms, seed development is controlled by the coordinated development of the embryo, endosperm and seed coat, while final seed volume relates predominantly to the maximum volume attained by the endosperm [107]. There are clear indicators that cytokinins are biosynthesized in the endosperm. However, the high levels of cytokinins in the seed coats of legume species was a surprise [101], clearly indicating a key point of control, which still requires further investigation.

The seed coat in leguminous species is typically a multi-layered structure, whereas in the cereal grain, in which the ovary wall is fused with the seed coat, the enlarged pericarp is considered to take over some of the key functions of the seed coat [153]. Nutrients passing from the mother plant to the endosperm and developing embryo must traverse the seed coat. Specialized transfer tissues, particularly evident in maize, which develop in a coordinated fashion on either side of the apoplast, direct and facilitate nutrient flow toward the growing endosperm and embryo [153].

The seed coat is clearly the key transit tissue for the flow of metabolites in legumes, but it appears to prevent xylem-supplied cytokinin from reaching the embryo, with the conclusion that xylem-derived cytokinins may exert little if any control over embryo development in legume seeds, which may be completely autonomous in terms of cytokinin [101]. A potential role for the cytokinins located in the seed coat is interacting with SWEETS and CWINV at the maternal–filial interface and to provide hexoses to drive cell division, metabolism and expansion of the endosperm. A reduction in CWIN activity is considered a marker for the transition from morphogenesis to maturation and storage [9,105]. Once cell division and differentiation are completed in the endosperm and embryo, it could be that the cytokinins have no role within the cotyledons until germination. The CKX-resistant nature of the accumulated cytokinins late in development would suggest this.

In cereals, more attention needs to be paid to the maternal tissues surrounding the endosperm during the various developmental stages to determine the site(s) of synthesis of the cytokinin. Are the endosperm and the embryo autonomous for cytokinin? If not, and if cytokinin is indeed supplied by the maternal tissues during grain fill [102], where is its site of biosynthesis and what is, or are, its translocation pathway(s) within the tissues of the grain? Notably, across both the pre-fill and fill phases of seed development, SWEET transporters fully account for maternal sucrose efflux in cereals [9].

### 4.4. Conclusions

Current evidence points to two, possibly three, distinctive roles played by the cytokinins in the developing seed: firstly, that of directing cell division (and therefore being a determinant of sink capacity), with a specific role in the initiation of cellularisation [11,142,152]; secondly, that of being a facilitator in the transfer of metabolites to the endosperm during the morphogenesis phase of seed development (and thereby playing a role in sink strength); and thirdly, a potential role associated with the accumulation of storage reserves. With an awareness of the complex nature of the cytokinin profile in seed tissues, but with new genes (*CPN1, CYP735A3* and *4*) emphasising the critical importance of *t*Z, hopefully new research will help to resolve the role(s) that cytokinins play within the developing seed.
